# Insights into the pathogenesis of herpes simplex encephalitis from mouse models

**DOI:** 10.1007/s00335-018-9772-5

**Published:** 2018-08-23

**Authors:** Mathieu Mancini, Silvia M. Vidal

**Affiliations:** 1 Department of Human Genetics, McGill University, Montreal, QC Canada; 2 McGill Research Centre on Complex Traits, McGill University, 3649 Promenade Sir William Osler, Montreal, QC H3G 0B1 Canada

## Abstract

A majority of the world population is infected with herpes simplex viruses (HSV; human herpesvirus types 1 and 2). These viruses, perhaps best known for their manifestation in the genital or oral mucosa, can also cause herpes simplex encephalitis, a severe and often fatal disease of the central nervous system. Antiviral therapies for HSV are only partially effective since the virus can establish latent infections in neurons, and severe pathological sequelae in the brain are common. A better understanding of disease pathogenesis is required to develop new strategies against herpes simplex encephalitis, including the precise viral and host genetic determinants that promote virus invasion into the central nervous system and its associated immunopathology. Here we review the current understanding of herpes simplex encephalitis from the host genome perspective, which has been illuminated by groundbreaking work on rare herpes simplex encephalitis patients together with mechanistic insight from single-gene mouse models of disease. A complex picture has emerged, whereby innate type I interferon-mediated antiviral signaling is a central pathway to control viral replication, and the regulation of immunopathology and the balance between apoptosis and autophagy are critical to disease severity in the central nervous system. The lessons learned from mouse studies inform us on fundamental defense mechanisms at the interface of host–pathogen interactions within the central nervous system, as well as possible rationales for intervention against infections from severe neuropathogenic viruses.

## Introduction

Herpes simplex virus types 1 and 2 (HSV-1 and HSV-2) are among the most common human pathogens. These viruses can establish life-long latency, such that an estimated 3.7 billion people under age 50 (67%) have been infected with HSV-1 and 417 million people aged 15–49 (11%) with HSV-2, worldwide (Looker et al. 2015a). There are important geographical differences however, where HSV-1 is universal in the developing world and in the United States, and HSV-2 is most prevalent in Africa (Looker et al. 2015b). HSV-2 disproportionally affects women and increases the risk of acquiring the human immunodeficiency virus (McQuillan et al. 2018). HSV infections are generally confined to the oro-labial and genital skin and mucosa, although the viruses are the most frequent cause of sporadic encephalitis (Looker et al. 2017; Steiner and Benninger 2013), a potentially deadly infection of the central nervous system (CNS). The characteristic clinical presentation of HSV encephalitis usually consists of fever, seizures, and often focal or generalized neurological deficits depending on the clinical form, namely neonatal HSV or herpes simplex encephalitis (HSE). In neonatal HSV, brain involvement is generalized, and the usual cause is HSV-2 (Long et al. 2011), which is acquired at the time of delivery in incident mothers. Post-partum infection is thought to be acquired by contact with HSV-1 shed by caregivers (10% of cases). HSV-1 is the predominant cause of HSE, with 10% of the cases caused by HSV-2 (Solomon et al. 2012). HSE typically affects the frontal and temporal lobes (Kaewpoowat et al. 2016), but in rare cases, the brainstem may be preferentially involved (Livorsi et al. 2010). The age-specific incidence is bimodal, with about 1/3 of cases observed in children between 3 months and 20 years, and the other in adults over 60, representing approximately 2/3 of the cases. Before the advent of acyclovir and other antiviral therapies, the mortality rate associated with central HSV infection was 70%. Treatment is effective if started promptly; in contrast, delays in treatment can be devastating. It is striking that in HSE, which still has a mortality approaching 30% and causes serious brain damage, there have been no significant enhancements of therapy in the last 30 years (Haubenberger and Clifford 2016). Improved tools are needed to treat, diminish the risk of, and prevent HSE based on a molecular understanding of HSE disease mechanisms.

Although there have been important advances in understanding disease neuropathogenesis [reviewed in Koyuncu et al. (2013), Kramer and Enquist (2013)], the precise mechanisms leading to HSE are not known. The virus enters the body by infecting epithelial or mucosal cells in peripheral tissues before entering sensory neurons. The virus genome reaches the soma of the sensory neuron as an episome that expresses a single transcript, LAT, and several microRNAs. The LAT RNA counteracts apoptosis of the neuronal cell and allows maintenance of the viral genome (Ahmed et al. 2002). The virus also has mechanisms (ICP34.5) that counteract autophagy, another antiviral mechanism in neurons (Orvedahl et al. 2007). During active replication, the virus expresses proteins (ICP0) that block innate responses mediated by type I interferon (IFN I), preventing the activation of the innate antiviral response (Lin et al. 2004). If the infection is not rapidly controlled, inflammatory responses are triggered and IFNs activate NK cell and T cell responses that are critical to clearance but also need to be tightly controlled to prevent inflammatory damage in the CNS (Menendez and Carr 2017). Pathological studies show that HSE is associated with virus replication, neuronal cell death, and the presence of inflammatory infiltrates; however, the relative contribution of each response to pathology or defense is not entirely clear (Michael et al. 2016; Wnek et al. 2016). Also outstanding is the question of which cellular and viral determinants can restrict replication at mucosal sites of infection, or permit the rare escape of virus to the CNS.

Host and viral genetic factors may interact to cause variability of herpesvirus neurovirulence. Genetic differences in isolates from HSE patients and oro-labial infections indicated that at least half of the cases of HSE are caused by a different viral strain than the one responsible for cold sores in the same individual, suggesting that HSE is due to primary infection rather than reactivation (Steiner 2011). Advancements in high-throughput sequencing have now made it possible to study the full extent of genetic variation in the viral population within an infected host, or that being transmitted between different hosts (Pandey et al. 2017; Parsons et al. 2015), and to provide the potential molecular basis for neurovirulence.

In recent years, the clinical genetic study of patients and families with HSE has revealed that single-gene inborn errors of innate or cell-intrinsic immunity can underlie enhanced susceptibility to specific viral infections in otherwise healthy individuals. Unique aspects of HSE molecular pathology have also been clarified with the advent of next-generation sequencing technologies. Thus, it was shown that HSE in two children was the consequence of an autosomal recessive deficiency in the intracellular protein UNC93B1 leading to impaired cellular IFN I responses (Casrouge et al. 2006). Subsequent work has confirmed that HSE in children may result from single-gene errors in Toll-like receptor 3 (TLR3)-IFN I pathways (Herman et al. 2012; Perez de Diego et al. 2010; Sancho-Shimizu et al. 2011; Zhang et al. 2007). These genetic defects all lead to reduced IFN I induction in patient cells, upon HSV-1 infection or ex vivo stimulation of the TLR3 pathway, in both fibroblasts and specific CNS cell lineages derived from induced pluripotent cells from the patient (Lafaille et al. 2012). Whereas defects in the TLR3 pathway are remarkably specific for manifestations of HSE caused by HSV-1, some patients have immunodeficiencies to various infectious phenotypes. Patients with susceptibility to herpes and mycobacterial infections have mutations in the transcription factor STAT1 and in NF-κB essential modulator NEMO (Audry et al. 2011; Dupuis et al. 2003). More recently, several patients with brainstem-localized HSV-1, norovirus, or influenza virus infections were found to lack intrinsic antiviral immunity due to mutations in the RNA binding protein, DBR1 (Zhang et al. 2018). Why such genetic determinants should manifest in childhood, but not in adult HSE, is unclear.

These groundbreaking discoveries (recently reviewed in (Casanova 2015; Zhang et al. 2013b)) have clarified the critical protective role of innate IFN responses that are non-redundant in the immune system. Specific mutations in components of the innate response account only for a minority of patients and fail to explain completely the different disease manifestations in the newborn HSV, childhood HSE, and the elderly population. Four decades ago, Lopez et al. reported that the infection of inbred mouse strains with HSV-1 mimicked dramatic differences in the presentation of encephalitis and survival observed in humans, justifying the search of genetic determinants of pathogenesis in mice (Lopez 1975). Whereas the phenotypes of human mutations have been recapitulated in mouse models for the most part, studies in mice have led to the discovery of new cell-intrinsic (Table 1) and immune cell-mediated (Table 2) disease mechanisms. This review will focus on how mouse models have contributed to our understanding of HSE and on the contributions of host genetics and of *Trl3*-dependent and independent mechanisms to HSV antiviral immunity. We also discuss future perspective on how these discoveries may lead to the development of improved therapies tailored to specific forms of human HSE.

**Table 1:** Gene deficiencies affecting cell-intrinsic responses to HSV encephalitis in mice, and to other infections in humans

Targeted allele(s)	Protein	Function	Survival phenotype^†^	Virus	Infection route	References	Human gene	Infectious agent or disease	OMIM number^§^
*Pvrl1* ^−/−^	Nectin-1	Cell-surface HSV entry receptor	Resistant	HSV-2	Intracranial	Kopp et al. (2009)			
*Ifnar* ^−/−^	IFNα/βR1	Type I IFN receptor	Susceptible	HSV-1	Intracranial	Wang et al. (2012)	*IFNAR2*	Disseminated vaccine measles, HHV-6	602376
			Susceptible	HSV-2	Intravaginal	Lee et al. (2017), Reinert et al. (2012)			
*Tlr3* ^−/−^	TLR3	Endosomal pattern recognition receptor	Susceptible	HSV-2	Intravaginal	Reinert et al. (2012)	*TLR3*	HSV-1	603029
*Ticam1* ^−/−^	TRIF	TLR3 cascade adaptor protein	Susceptible*	HSV-1	Intranasal	Menasria et al. (2013)	*TICAM1*	HSV-1, HSV-2	607601
		STING cascade adaptor protein	Susceptible*	HSV-1	Corneal	Wang et al. (2016)			
*Tlr4* ^−/−^	TLR4	Cell-surface pattern recognition receptor	As WT	HSV-1	Intraperitoneal	Kurt-Jones et al. (2004)			
*Tlr2* ^−/−^	TLR2	Cell-surface pattern recognition receptor	As WT	HSV-1	Intranasal	Lima et al. (2010)			
			Resistant	HSV-1	Intraperitoneal	Kurt-Jones et al. (2004)			
			Resistant	HSV-1	Intracranial	Wang et al. (2012)			
*Tlr9* ^−/−^	TLR9	Endosomal pattern recognition receptor	Susceptible*	HSV-1	Intranasal	Lima et al. (2010)			
			As WT	HSV-1	Intracranial	Wang et al. (2012)			
*Tlr2* ^−/−^ *Tlr9* ^−/−^	TLR2/9	See above	Susceptible	HSV-1	Intranasal	Lima et al. (2010)			
			As WT	HSV-1	Intracranial	Wang et al. (2012)			
*Unc93b1* ^3 *d*^	UNC93B1	TLR cascade adaptor protein	As WT	HSV-1	Intracranial	Wang et al. (2012)	*UNC93B1*	HSV-1	608204
*Myd88* ^−/−^	MYD88	TLR cascade adaptor protein	Susceptible	HSV-1	Intranasal	Mansur et al. (2005)	*MYD88*	Bacterial (*pyogenes*)	602170
			Resistant	HSV-1	Intravenous	Honda et al. (2005)			
*Mb21d1* ^−/−^	cGAS	Cytosolic DNA sensor	Susceptible	HSV-1	Corneal	Reinert et al. (2016)			
*Sting* ^*gt*/ *gt*^	STING	Cytosolic DNA sensor	Susceptible	HSV-1	Corneal	Reinert et al. (2016)			
*Sting* ^−/−^	STING	IFN I-dependent autophagy	As WT	HSV-1	Corneal	Parker et al. (2015)			
			Susceptible	HSV-1	Intravenous/intracranial	Parker et al. (2015)			
*Trim14* ^−/−^	TRIM14	Modifier of cGAS	Susceptible*	HSV-1	Intravenous	Chen et al. (2016)			
*Usp13* ^−/−^	USP13	Modifier of STING	Resistant	HSV-1	Intravenous	Sun et al. (2017a)			
*Usp21* ^*fl*/ *fl*^ *Lyz2-cre*	USP21	Modifier of STING	Resistant	HSV-1	Intravenous	Chen et al. (2017)			
*Rhbdf2* ^−/−^	iRhom2	STING signaling cascade	Susceptible	HSV-1	Intravenous	Luo et al. (2016)			
*Mavs* ^−/−^	IPS-1 (MAVS)	RIG-I/MDA5 cytosolic RNA sensing pathway	As WT	HSV-1	Intranasal	Menasria et al. (2013)			
*Irf3* ^−/−^	IRF3	IFN signaling transcription factor	Susceptible*	HSV-1	Corneal	Murphy et al. (2013)	*IRF3*	HSV-1	616532
			As WT	HSV-1	Intravenous	Honda et al. (2005)			
*Irf7* ^−/−^	IRF7	IFN signaling transcription factor	Susceptible	HSV-1	Corneal	Murphy et al. (2013)	*IRF7*	Severe influenza disease	605047
			Susceptible	HSV-1	Intravenous	Honda et al. (2005)			
*Irf3* ^−/−^ *Irf7* ^−/−^	IRF3/7	See above	Susceptible	HSV-1	Corneal	Murphy et al. (2013)			
*Rnf128* ^−/−^	RNF128	Modifier of TBK1	Susceptible	HSV-1	Intravenous	Song et al. (2016)			
*Hcfc2* ^*fls*/ *fls*^	HCFC2	Facilitates IRF1/IRF2 binding to *Tlr3* promoter	Susceptible	HSV-1	Retro-orbital	Sun et al. (2017b)			
*Stat1* ^−/−^	STAT1	IFN signaling transcription factor	Susceptible	HSV-1	Corneal	Katzenell et al. (2014)	*STAT1*	Mycobacteria	614892
								HSV-1, EBV, VZV	613796
								Candidiasis	614162
*Isg15* ^−/−^	ISG15	Interferon-stimulated gene	Susceptible*	HSV-1	Intracranial/ corneal	Lenschow et al. (2007)	*ISG15*	Mycobacteria	147571
*Oasl1* ^−/−^	OASL1	Interferon-stimulated gene	Resistant	HSV-2	Intravaginal	Oh et al. (2016)			
*Trp53* ^−/−^	p53	Regulator of cellular stress	Resistant	HSV-1	Intracranial	Maruzuru et al. (2016)			

*Incomplete penetrance of survival phenotype. These gene-deficient animals, although more susceptible than WT controls, do not all succumb to HSV infection

^†^Respectively, “resistant” and “susceptible” denote reduced or increased survival to HSV infection, as compared to WT control mice. “As WT” describes gene-deficient animals that are equally susceptible or resistant to HSV infection as WT controls

^§^Reference to human gene deficiencies available on the Online Mendelian Inheritance in Man (OMIM) database (http://www.omim.org)

**Table 2:** Gene deficiencies affecting cell-mediated responses to HSV encephalitis in mice, and to other infections in humans

Targeted allele(s)	Protein	Function	Survival phenotype^†^	Virus	Infection route	References	Human genes	Infectious agent or disease	OMIM number^§^
*Ifnar* ^−/−^ *Ifngr* ^−/−^	IFN-αβγR	IFN I and IFN II receptors	Susceptible	HSV-1	Corneal	Parker et al. (2016)			
*Irf9* ^−/−^	IRF9	IFN signaling transcription factor	Susceptible	HSV-2	Intravaginal	Lee et al. (2017)			
*Il15* ^−/−^	IL-15	T and NK cell proliferation	Susceptible*	HSV-2	Intravaginal	Ashkar and Rosenthal (2003)			
*Rag1* ^−/−^	RAG-1	B and T cell development	Susceptible	HSV-1	Intranasal/cutaneous	Milora et al. (2017), Zolini et al. (2014)	*RAG1*	T- B- NK + deficiency, HCMV	609889
*Rag2* ^−/−^	RAG-2	B and T cell development	Susceptible	HSV-1	Corneal	Ramakrishna and Cantin (2018)	*RAG2*	T- B- NK + deficiency, respiratory infections	233650
*Rag2* ^−/−^ *Il2rg* ^−/−^	RAG-2, γc (CD132)	B, T, and NK cell development	Susceptible	HSV-2	Intravaginal	Ashkar and Rosenthal (2003)	*IL2RG*	T- B + NK- deficiency, EBV	308380
*Ighm* ^−/−^	Ig heavy chain μ	Component of B cell receptor	Susceptible	HSV-1	Intraperitoneal	Beland et al. (1999)	*IGHM*	B- deficiency, severe bacterial infections	147020
*B2m* ^−/−^	β2M	Component of MHC class I receptor	Susceptible	HSV-1, HSV-2	Cutaneous	Holterman et al. (1999), Manickan and Rouse (1995)	*B2M*	CD8^+^ T- deficiency, impaired T/NK cytotoxicity, sinopulmonary infections	109700
*Cd4* ^−/−^	CD4	CD4^+^ T cell development	Susceptible	HSV-1, HSV-2	Cutaneous	Manickan and Rouse (1995)			
*Cd8* ^−/−^	CD8	CD8^+^ T cell development	Susceptible	HSV-1	Intranasal	Zolini et al. (2014)	*CD8*	CD8^+^ T deficiency, recurrent bacterial (*H. influenzae*) infections	186910
*Ptprc* ^*L*3 *X*^	CD45	Lymphoid cell development	Susceptible	HSV-1	Intraperitoneal	Caignard et al. (2013)	*PTPRC*	T- NK- deficiency	151460
*Lta* ^−/−^	LTα	TNF family cytokine	Susceptible	HSV-1	Intramuscular	Kumaraguru et al. (2001)			
*Ifngr* ^−/−^	IFNγR	IFN II receptor	Susceptible*	HSV-1	Corneal	Cantin et al. (1999)	*IFNGR1*	Mycobacteria and *Salmonella*	107470
							*IFNGR2*	Mycobacteria and *Salmonella*	147569
*Ifng* ^−/−^	IFNγ	Inflammatory cytokine	As WT	HSV-1	Corneal	Cantin et al. (1999)			
			Susceptible*	HSV-1	Corneal	Ramakrishna and Cantin (2018)			
			Susceptible*	HSV-1	Intranasal	Mansur et al. (2005)			
			Susceptible*	HSV-2	Intravaginal	Ashkar and Rosenthal (2003)			
*Socs2* ^−/−^	SOCS2	Suppressor of cytokine signaling	Resistant	HSV-1	Intracranial	da Cunha Sousa et al. (2016)			
*Il10* ^−/−^	IL-10	Inflammatory cytokine	Susceptible*	HSV-1	Corneal	Ramakrishna and Cantin (2018)			
*Il6* ^−/−^	IL-6	Inflammatory cytokine	Susceptible*	HSV-1	Corneal	LeBlanc et al. (1999)			
*Tnf* ^−/−^	TNFα	Inflammatory cytokine	Susceptible*	HSV-1	Intranasal/ corneal	Lundberg et al. (2007), Sergerie et al. (2007)			
*Il1b* ^−/−^	IL-1β	Inflammatory cytokine	Susceptible	HSV-1	Intranasal	Sergerie et al. (2007)			
*Tnf* ^−/−^ *Il1b* ^−/−^	TNFα IL-1β	See above	Susceptible	HSV-1	Intranasal	Sergerie et al. (2007)			
*Il36b* ^−/−^	IL-36β	Inflammatory cytokine	Susceptible*	HSV-1	Cutaneous	Milora et al. (2017)			
*Tnfrsf1a* ^−/−^	p55	TNF receptor subunit	As WT	HSV-1	Corneal	Lundberg et al. (2007)			
			Susceptible*	HSV-1	Corneal	Mohankrishnan et al. (2017)			
*Tnfrsf1a* ^−/−^ *Tnfrsf1b* ^−/−^	p55/p75	TNF receptor subunits	As WT	HSV-1	Corneal	Lundberg et al. (2007)			
*Nos2* ^−/−^	iNOS	Synthesis of nitric oxide	Susceptible	HSV-1	Intranasal	Zolini et al. (2014)			
*Ptafr* ^−/−^	PAFR	Platelet activating factor receptor	Resistant	HSV-1	Intracranial	Vilela et al. (2016)			
*Cxcl10* ^−/−^	CXCL10	Chemokine	Susceptible*	HSV-1	Corneal	Wuest and Carr (2008)			
*Cxcr3* ^−/−^	CXCR3	Chemokine receptor	Resistant	HSV-1	Corneal	Wickham et al. (2005)			
*Ccr5* ^−/−^	CCR5	Chemokine receptor	As WT	HSV-1	Intracranial	Vilela et al. (2013)	*CCR5* (32-BP DEL)	Resistance to HIV	601373
			Resistant	HSV-1	Corneal	Carr et al. (2006)			
*Cx3cr1* ^−/−^	CX3CR1	Chemokine receptor	Susceptible*	HSV-1	Intranasal	Menasria et al. (2017)			

*Incomplete penetrance of survival phenotype. These gene-deficient animals, although more susceptible than WT controls, do not all succumb to HSV infection

^†^Respectively, “resistant” and “susceptible” denote reduced or increased survival to HSV infection, as compared to WT control mice. “As WT” describes gene-deficient animals that are equally susceptible or resistant to HSV infection as WT controls

^§^Reference to human gene deficiencies available on the Online Mendelian Inheritance in Man (OMIM) database (http://www.omim.org)

## Mouse experimental models of HSV infection and HSE

HSV infections can be modeled experimentally in mice. Although humans are the principal natural reservoir for HSV, mice share several cell-surface receptors that allow for both systemic and neurotropic infection with human herpes viruses. These receptors include Nectin-1 (*Pvrl1*) and herpes virus entry mediator HVEM (*Tnfrsf14*), both expressed on epithelial keratinocytes and fibroblasts (Petermann et al. 2015a, b). In addition, nectin-1 expression on neurons can facilitate the entry of HSV; intracranially infected *Pvrl1*^−/−^ mice do not develop encephalitis, and lytic viral replication in severely limited in the CNS compared to WT mice (Haarr et al. 2001; Kopp et al. 2009). While the establishment of latent HSV infection in neurons of the trigeminal ganglia (TG) is well documented in mice, the spontaneous reactivation of latent virus is difficult to measure reliably in immunocompetent hosts, but can be triggered in vivo under stress, or ex vivo in explanted TG neuron cultures (Doll and Sawtell 2017; Matundan et al. 2016; Ramakrishna et al. 2015).

Different routes of infection recapitulate different aspects of HSV pathogenesis, with some routes better suited for investigating the peripheral host response. Cutaneous inoculation of the flank, footpad, or of the oral mucosa will often limit pathology and viral replication to the site of inoculation, and involves the early recruitment of neutrophils, NK cells, and antigen-presenting cells that will migrate to secondary lymphoid organs and prime the adaptive immune response (Milora et al. 2017). More commonly, intraperitoneal or intravenous inoculations are used to model systemic HSV infection, where viral replication may occur notably in the liver (Caignard et al. 2013; Chen et al. 2016; Parker et al. 2016). Intraperitoneal and intravenous models can also result in viral invasion of the CNS and lethal encephalitis, although many studies report lethality rather than HSV titers in brain tissue or infiltration of immune cells.

Other sites of delivery better approximate the natural course of primary infection observed in human patients. Intravaginal or intranasal inoculation, or intraocular delivery by corneal scarification, can each establish productive infection in the vaginal, nasal, and corneal epithelia, respectively (Menasria et al. 2017; Reinert et al. 2012, 2016). Through the contact of the nasal epithelium and eye with the sensory termini of TG neurons, HSV virions travel by retrograde axonal transport along the TG to invade the CNS (Cook and Stevens 1973). From here, the virus is typically localized to hindbrain (brainstem, cerebellum), and can be detected in the brain ependyma and lateral ventricles (Conrady et al. 2013; Kroll et al. 2014). However, mice rarely develop the temporal or frontal lobe-localized infected lesions that are characteristic of human childhood HSV encephalitis. The olfactory bulbs can also host lytic viral replication (Menasria et al. 2017), although the virus generally avoids them altogether in intranasal models (Shivkumar et al. 2013). Alternatively, intravaginal inoculation will lead to productive infection in the dorsal root ganglia, whereupon viral particles can reach the CNS via spinal cord neurons (Wang et al. 2013). While paracellular entry is also possible in most models—as pro-inflammatory cytokines and matrix metalloproteases weaken the tight junctions of the blood–brain barrier (BBB) (Sellner et al. 2006)—HSV cannot passively invade the CNS in the event of elevated systemic HSV replication (viremia), a common invasion mechanism in many kinds of arboviral encephalitis (Salimi et al. 2016). Finally, intracranial HSV inoculation is also frequently employed in mice, a route that bypasses retrograde axonal transport and often results in disseminated CNS viral replication (Wang et al. 2012).

Additional considerations for modeling HSE include the choice of HSV strain, which have different capacities for neurovirulence and CNS invasion. For HSV-1, highly neurovirulent strains including strain 17 and McKrae, and milder strains like KOS, are often used for experimental encephalitis in mice, whereas neurovirulent HSV-2 strains often result in meningitis (Bergstrom et al. 1990; Wang et al. 2013). Higher doses usually result in more severe pathology, but can be titrated in vivo to a level where susceptible controls succumb and resistant controls survive (Caignard et al. 2013). Other non-genetic factors include the age of mice at infection, where the severity and CNS viral invasion of HSV is greater in neonate mice than in adults (Wilcox et al. 2015).

As with human HSE, host genetics play an important role in mouse susceptibility to lethal encephalitis. Inbred strains of mice have shown differential susceptibility to HSV-1 infection, with C57BL/6 mice noted for their resistance compared to other strains such as A/J, BALB/c (Lopez 1975, 1980), or 129S6SvEv/Tac (Cantin et al. 1999), which are relatively susceptible to fatal infection. Forward genetic screens have been performed to identify new susceptibility loci or genes on resistant backgrounds (Caignard et al. 2013; Lundberg et al. 2003; Sun et al. 2017b), and significantly more reverse genetic studies have used single-gene knockout mice, or lymphopenic mice, to inform important mechanisms of host defense. Early targeted mutagenesis studies have made extensive use of embryonic stem cells obtained from 129S6SvEv/Tac mice, which as mentioned above are relatively susceptible to HSV-1 infection (Linder 2006). Hence to control for genetic background effects in these studies, the mutant allele has been serially back-crossed to HSV-resistant C57BL/6 to generate congenic mice. However, flanking gene differences between experimental animals, and between them and their controls, can present itself as variations in the degree of susceptibility (penetrance) of the phenotype. In establishing a hierarchy in the impact of the mutations, or for comparison between different laboratories, caution must be exerted in the interpretation of the results. To better guide the reader, we have thus indicated cases in which incomplete penetrance has been observed. Here, we will focus on groups of genes that together implicate cell-intrinsic and cell-mediated mechanisms, required in the periphery, in infiltrating hematopoietic immune cells, and in resident neural cells to assure a protective response to HSV-1 infection. Ultimately, these mouse models have helped to inform the genetics of HSE pathogenesis even beyond the TLR3/IFN I axis implicated in childhood human HSE.

## Cell-intrinsic IFN I responses to HSV in mice

**Fig. 1:**
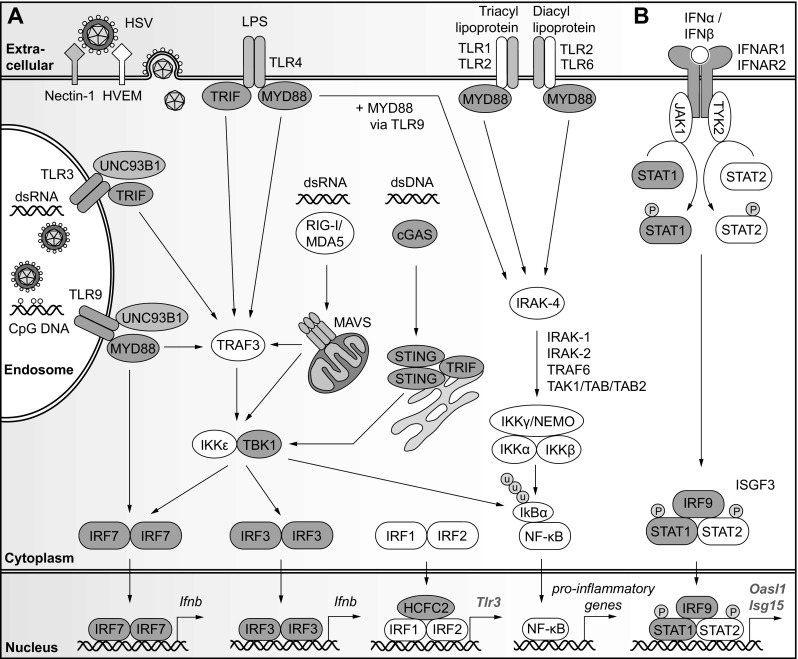
Selected genetic factors and pathways essential to the cell-intrinsic response to HSV encephalitis in mice. **a** Following infection and the entry of HSV via endosomes, or of HSV nucleocapsids into the cytoplasm, viral nucleic acids and other pathogen-associated molecular patterns are recognized by endosomal Toll-like receptors (including TLR3 and TLR9), TLR at the cell surface (including TLR1/2, TLR2/6 and TLR4), and by cytoplasmic DNA sensors cGAS/STING and RNA sensors RIG-I/MDA5. These pattern recognition receptors initiate signaling through factors including MYD88, TRIF, and TBK1 to promote the transcription of type I interferon (IFN I) and pro-inflammatory genes via transcription factors IFR3, IRF7, and NF-κB. **b** The subsequent production of IFNα and IFNβ and engagement of the type I interferon receptor (IFNAR1/2) initiates JAK/STAT signaling to promote the expression of antiviral interferon-stimulated genes (ISG) that establish a protective antiviral state in the infected cell. For each reported gene defect in mice, the encoded protein is color-coded above as follows: (1) red for gene defects that lead to HSV susceptibility in at least one mouse model, (2) blue for gene-deficient mice with equal or increased resistant compared to WT control mice, and (3) white for genes that have not been tested in mice. Further gene defects identified in mice that drive susceptibility or resistance to HSV infection including the cGAS modifier TRIM14, STING cascade modifiers USP13, USP21, and iRhom2, and TBK1 modifier RNF128 are detailed in Table 1. (Color figure online)

IFN I cytokines in mice consist of 13 isoforms for IFNα and one for IFNβ, and together ensure the control and restriction of viral replication in various cell types (Pestka et al. 2004). For DNA viruses like HSV, IFN I signaling can be initiated downstream of endosomal TLRs upon their recognition of foreign viral nucleotides (TLR3 recognizing double-stranded RNA, TLR8/7 recognizing single-stranded RNA, and TLR9 recognizing unmethylated cytosine-phosphate-guanine, or CpG, DNA). Furthermore, cytosolic viral DNA will be sensed by the cyclic GMP-AMP synthase/stimulator or interferon genes (cGAS/STING) pathway. On the other hand, RIG-I/MDA5-mediated recognition of cytosolic double-stranded RNA appears to have a more limited role in anti-HSV immunity (Liu et al. 2016; Menasria et al. 2013). IFNγ-inducible protein 16 (IFI16) is also a major viral DNA sensor in humans, with some evidence suggesting that IFI200-family genes (including *Ifi204* and *Ifi205*) respond to IFN I or infection to fulfill a homologous role in mice (Ghosh et al. 2017; Hertel et al. 1999). The above signaling cascades converge notably on NF-κB and interferon regulatory factor (IRF) family transcription factors to modulate IFN I expression and other inflammatory cytokines. Upon binding to the IFN I receptor (IFNAR, including IFNAR1 and IFNAR2 subunits), IFN I stimulates the transcription of various interferon-stimulated genes (ISGs) that help to maintain a global antiviral program in the cell. Thus, *Ifnar*^−/−^ mice are susceptible to lethal intracranial HSV-1 (Wang et al. 2012) and to intravaginal HSV-2 (Lee et al. 2017; Reinert et al. 2012). Mouse models that are deficient for discrete factors involved in IFN I signaling have further defined the role of TLR and cGAS/STING viral nucleotide sensing in the development of HSE (Table 1; Fig. 1).

### TLR signaling

Most genetic aberrations that drive HSE in children fit into the TLR3/IFN I axis, yet mice have broadened our understanding of innate viral sensing in the HSV-infected CNS to include additional TLRs. In glial cell cultures, murine astrocytes can upregulate TLR2, TLR3, and TLR4 after interacting with activated microglia, which themselves express most TLRs at steady-state and upon activation (Holm et al. 2012; Rosenberger et al. 2014). Following intravaginal HSV-2 infection, *Tlr3*^−/−^ mice are highly susceptible to HSE, and show increased leukocyte infiltration, viral load, and infection of astrocytes and neurons in the CNS (Reinert et al. 2012). However, while global IFN I production was unaffected in the *Tlr3*^−/−^ CNS, astrocytes from these animals failed to produce IFNβ in response to HSV-2 and were thus more readily infected ex vivo. Further, TRIF (*Ticam1*^−/−^) null mice, lacking the TLR3-specific intracellular adaptor TRIF, are partially susceptible to HSE, where decreased IFNβ production at day 5 post intranasal HSV-1 infection was associated with 60% of mice developing lethal encephalitis by day 8 (Menasria et al. 2013). Thus, the murine TLR3 cascade may contribute to protective CNS IFN I production, as observed in many human childhood HSE patients.

Other TLRs, including those that depend on downstream intracellular adaptor protein MYD88, are required for protective responses to HSV. Upon intranasal HSV-1 challenge, *Tlr2*^−/−^ mice are resistant to HSE, and *Tlr9*^−/−^ or double *Trl2*^−/−^*Tlr9*^−/−^ knockout mice are partially and fully susceptible, respectively (Lima et al. 2010; Mansur et al. 2005). In this model, lesions and cellular infiltrate occur in the brain of HSE-susceptible mice. Yet, *Tlr2* and *Tlr9* gene expression is mostly upregulated in the TG after infection in resistant WT mice (Zolini et al. 2014), suggesting that the TG is a crucial checkpoint for viral recognition and control. Lower levels of CCL2 have also been reported in HSE-resistant *Tlr2*^−/−^ mice, suggesting that the receptor may also play an auxiliary role in HSE pathology (Kurt-Jones et al. 2004). However, using a more acute intracranial HSV-1 infection model to bypass the TG altogether, *Tlr9*^−/−^, *Trl2*^−/−^*Tlr9*^−/−^ and adaptor *Unc93b1*-null mutant mice are only as susceptible as C57BL/6 mice, and each with normal IFNβ expression in the brain (Wang et al. 2012); here *Tlr2*^−/−^ mice are significantly more resistant than WT. Similarly, *Myd88*^−/−^ mice are partially susceptible following intranasal inoculation (Mansur et al. 2005), but fully resistant to systemic HSV-1 infection (Honda et al. 2005). Overall, proper viral sensing via these MYD88-dependent TLRs is required in the TG to mount a protective innate response to CNS invasion of HSV, but may be dispensable when the virus is introduced directly in the brain parenchyma or in the periphery.

### cGAS/STING signaling

Recent studies in mice have uncovered an important role for cytosolic viral DNA sensing in innate immunity to HSE. In infected mammalian cells, as HSV capsids are ubiquitinated and targeted to the proteasome for degradation, HSV genomic DNA may become available in the cytosol to bind cyclic GMP-AMP synthase (cGAS) (Horan et al. 2013). Newly produced cyclic GMP-AMP molecules can interact with and activate stimulator of interferon genes (STING), notably leading to downstream IFN I production. Accordingly, mice lacking either cGAS or STING are highly susceptible to HSE, and are characterized by elevated viral titers in the TG, brainstem, and further dissemination into the whole brain (Reinert et al. 2016). In particular, STING is required by infected microglia cells to produce IFN I and limit viral replication (Reinert et al. 2016), but has also been implicated in autophagy pathway-mediated viral clearance following systemic and intracranial HSV-1 infection (Parker et al. 2015).

Furthermore, modifiers of cGAS and STING function contribute to HSV-1 susceptibility in single-gene or tissue-specific knockout models. First, the stability and activation of cGAS is required to initiate effective antiviral responses. IFN I-induced expression of TRIM14 favors the recruitment of USP14, a deubiquitinase which in turn promotes further IFN I expression by modifying and protecting cGAS from autophagy-mediated degradation; thus, *Trim14*^−/−^ mice are more susceptible to infection (Chen et al. 2016). Second, deubiquitination of STING on different residues can improve or exacerbate pathology. Both *Usp13*^−/−^ mice (Sun et al. 2017a) and myeloid cell compartment-specific *Usp21*^*fl*/*fl*^ null mice (Chen et al. 2017) are more resistant to HSV-1 infection due to heightened IFN I signaling occurring in the absence of these STING deubiquitinases. Yet, STING can also be stabilized by the site-specific removal of ubiquitin chains. As reported in *Rhbdf2*^−/−^ mice, the failed iRhom2-dependent recruitment of deubiquitinase EIFS35 and adaptor TRAFβ to the STING complex, factors that normally stabilize STING and promote its transport to the endoplasmic reticulum, results in lower serum IFN I and increased HSV-1 susceptibility (Luo et al. 2016). Finally, it has been suggested that mice lacking TRIF, previously noted for their TLR3-dependent susceptibility to intranasal HSV-1 infection (Menasria et al. 2013), may otherwise produce lower IFN I due to a deficiency in STING signaling in a susceptible model of corneal infection (Wang et al. 2016). Here, an interaction with TRIF supports the activation and dimerization of STING, lowering viral replication in ex vivo infected primary *Ticam1*^−/−^ cells. Thus, TRIF is an example of a factor that bridges multiple innate sensing pathways.

### Transcription factors and IFN-stimulated genes

Viral molecular pattern sensors signal through downstream transcription factors that drive the expression of IFN I and the subsequent expression of ISGs. Among these transcription factors, the nuclear translocation of active IRF3 dimers leads to early IFNβ and IRF7 expression. In turn, active IRF7 promotes elevated IFNα, IFNβ, and ISG expression. Without both transcription factors, *Irf3*^−/−^*Irf7*^−/−^ mice cannot mount an antiviral response and succumb fully to corneal HSV-1 infection (Murphy et al. 2013). In this model, *Irf7* contributes most to the protective response, while *Irf3*^−/−^ mice are partially susceptible to infection. Furthermore, IRF3 phosphorylation and downstream IFN production are dependent on TBK1 kinase activity; positive regulation of TBK1 by E3 ubiquitin ligase RNF128 confers some protection to systemic HSV-1 infection in *Rnf128*-sufficient mice (Song et al. 2016). Additionally, other IRF-family members including IRF1 and IRF2 are known to modulate the transcription of *Tlr3*. In this context, mice carrying a chemically induced null mutation in *Hcfc2*, a factor that facilitates the interaction of IRF1 and IRF2 with the *Tlr3* promoter, show defects in TLR3-dependent IFN production and are susceptible to retro-orbital HSV-1 infection (Sun et al. 2017b).

Signal transducer and activator of transcription 1 (STAT1) is another key transcription factor in the response downstream of IFNAR signaling. Two *Stat1* knockout mouse models are commonly used, one with an N-terminal deletion (*Stat1*^−/−^ ΔNTD) that disrupts active complexes of phosphorylated STAT1 with phosphorylated STAT2 (Meraz et al. 1996), and another with a deletion in the DNA-binding domain (*Stat1*^−/−^ ΔDBD) (Durbin et al. 1996). Although the DNA-binding domain plays a more prominent role in the mounting of an adequate response to IFNβ and control of ex vivo infection, both *Stat1*^−/−^ models are highly sensitive to corneal HSV-1 infection and succumb by day 8 post-infection (Katzenell et al. 2014). Yet, HSV-1-infected *Stat1*^−/−^ ΔDBD and ΔNTD mice distinguish themselves by their respective systemic or CNS viral tropisms (Pasieka et al. 2011b). Upon ocular infection of *Stat1*^−/−^ ΔNTD mice that succumb to CNS-localized infection, active STAT3 and IL-6 drive inflammation in the corneal epithelium (Pasieka et al. 2009) before a heightened antiviral and pro-inflammatory response develops in the brainstem (Pasieka et al. 2011a). In these brainstems collected at the day 5 peak of HSV-1 replication, functional gene expression analysis revealed that IFN-I-dependent ISGs and IRF-family genes are upregulated in resistant 129S-background control animals; susceptible *Stat1*^−/−^ mice show increased expression of inflammatory chemokines (*Cxcl10, Ccl2*), cytokines (*Ifng, Il6, Il1b*), markers of cell infiltration (*Icam1, Il8rb*), and matrix metalloproteases (*Mmp3, Mmp8, Mmp9*), all typical of acute HSE pathogenesis (Pasieka et al. 2011a). Thus, STAT1-dependent IFN I signaling is pivotal for the initiation of a protective innate antiviral response to HSV-1 in the mouse CNS.

Certain ISGs have also been found to contribute to a protective antiviral response to HSV infection in mice. Notably, the lack of ISG15—an IFN I-induced ubiquitin-like factor that modifies several protein targets that help to establish an antiviral state in infected cells—renders mice more sensitive to lethal HSV-1 following intracranial and intraocular infections (Lenschow et al. 2007). On the other hand, negative regulators of IFN I, including IRF7 antagonist OASL1, can exacerbate viral pathogenesis; *Oasl1*^−/−^ mice are partially protected from lethal HSV-2 infection (Oh et al. 2016). Thus, genetic defects that target IFN I-responsive proteins, as well as components of viral pattern recognition pathways and their downstream transcription factor-dependent signaling, modify the innate capacity of mouse cells to reduce HSV-1 viral replication, and to ultimately clear the infection in vivo.

## Cell-mediated responses to HSV infection in mice

**Fig. 2:**
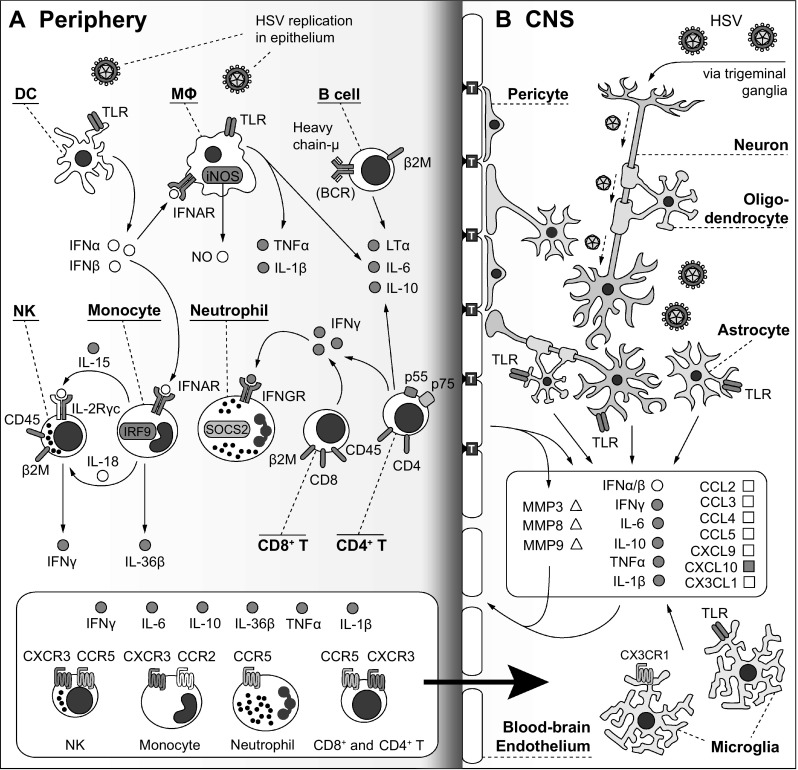
Selected genetic factors and pathways essential to the cell-mediated immune response to HSV encephalitis in mice. **a** HSV will first infect and replicate in epithelial cells and keratinocytes, activating chemokine pathways and triggering dendritic cells (DC) to produce type I interferon (IFN I). Responding to IFN I, macrophages (MΦ) and monocytes will produce inflammatory cytokines (TNFα, IL-1β), and contribute to the activation of cytotoxic IFNγ-producing natural killer cells (NK). Furthermore, activated B cells, and especially effector CD4^+^ T cells and cytotoxic CD8^+^ T cells, will help to maintain an adequate antiviral response including T cell-dependent production of cytokines like IFNγ, which will promote neutrophil expansion. While single-gene defects in factors including CD45, β2M, Heavy chain-μ, LTα, CD4, CD8, RAG-1, RAG-2, and IL-2Rγc have all been implicated in susceptibility to HSV encephalitis in mice, it remains unclear whether their essential function in immune cell development, or any specific effector functions, strictly drives susceptibility to infection. **b** In the CNS, the principal route of entry of HSV is via the axons of the trigeminal ganglia. HSV readily infects neurons, as well as glia including oligodendrocytes, astrocytes, and microglia. The TLR-dependent recognition of HSV by glia and neurons may drive expression of cytokines including IFN I, IFNγ, IL-6, TNFα, and IL-1β. Along with matrix metalloproteases (MMP3, 8, 9), these CNS cytokines and those produced in the periphery may disrupt the blood–brain barrier (BBB) by weakening the tight junctions (marked above by “T”-labeled blue squares) between BBB endothelial cells. Finally, the expression of various chemokines by CNS-resident cells will attract immune cells with cognate chemokine receptors (CCR2/CCL2; CCR5/CCL3, CCL4, CCL5; CXCR3/CXCL9, CXCL10; CX3CR1/CX3CL1) across the permeable BBB into the CNS (marked by the black arrow between panels **a** and **b**). In the CNS, these infiltrating immune cells may enhance virus clearance, but also contribute to HSE pathology. The pathways shown above have been directly involved in mouse HSE studies, but do not reflect the full production of factors by all immune, neuronal, and glial cells, or their complete downstream effects. For each reported gene defect in mice, the encoded protein is color-coded above as follows: (1) red for gene defects that lead to HSV susceptibility in at least one mouse model, (2) blue for gene-deficient mice with equal or increased resistant compared to WT control mice, and (3) white for genes that have not been tested in mice. (Color figure online)

In the context of neurotropic viral infection, immune cell populations contribute both to inflammation and to the recognition and targeted killing of infected cells. Spurred on by the production of antiviral factors, the protective cellular response to HSV-1 in mice progresses through three general phases. First, in the periphery, natural killer cells (NK), myeloid cells, and various dendritic cell (DC) subsets will produce IFN I, IFNγ, and other pro-inflammatory cytokines necessary for the further activation and proliferation of NK cells (Kassim et al. 2006; Swiecki et al. 2013). Antigen-presenting cells including DCs will migrate to the secondary lymphoid organs to expose B cells, CD4^+^ and CD8^+^ T cells to viral antigen. Next, these activated cytokine-producing lymphocytes and myeloid cells can invade the CNS by the lymphatics network or directly across an inflammation-weakened BBB. Ultimately, alongside activated glial cells, the CNS infiltration of immune cell can either benefit the host by eliminating infected cells and reducing viral titers, or drive overwhelming inflammation characteristic of HSE pathology. Mouse models have been especially useful to dissect the role of discrete genes in these three aspects of cell-mediated HSV-1 immunity (Table 2; Fig. 2).

### Peripheral lymphoid and myeloid cell responses

Innate IFN production plays a fundamental role in the activation of the cellular immune response to early HSV-1 replication. Infected *Ifnar*^−/−^*Ifngr*^−/−^ mice, lacking both type I and II IFN receptors, exhibit high viral loads in the liver due to a failure of the IFN signaling-deficient hematopoietic compartment to control the infection (Parker et al. 2016). For example, HSV-2 replication at the vaginal mucosa is controlled by the IFN I-dependent recruitment of cytotoxic Granzyme B^+^/IFNγ^+^ NK cells and CCL2-producing Ly6C^HI^ inflammatory monocytes; *Ifnar*^−/−^ mice recruit only CXCL1/CXCL2-producing Ly6C^LOW^ monocytes that instead promote neutrophil invasion, and fail to initiate a protective antiviral response (Uyangaa et al. 2015). In a similar model, *Ifnar*^−/−^ and *Irf9*^−/−^ mice are both more sensitive to lethal HSV-2 infection, where few IL-18-producing inflammatory monocytes are recruited to the vaginal mucosa, resulting in low IL-18-dependent production of IFNγ in NK cells (Lee et al. 2017). Lower levels of IFNγ are also implicated in an HSV-2-susceptible *Il15*^−/−^ model (Ashkar and Rosenthal 2003). As for the role of cytotoxic NK cells in the infected CNS, a forward genetics approach identified a locus on murine chromosome 6 that may underlie resistance to HSE in C57BL/6 mice (Kastrukoff et al. 2015). Susceptible BALB/c background mice, or NK-depleted C57BL/6 mice, exhibited viral spread to the cerebellum and augmented titers in the brainstem. Thus, NK-specific genes in this resistance locus, including *Cd94*, the Ly49 cluster, and NKG2 cluster, may contribute to the protective antiviral response to HSE.

Classical adaptive immune cells are also important in the control of acute HSV infection and the development of HSE in mice. Both *Rag*^−/−^ (lacking mature B and T cells) and *Rag2*^−/−^*Il2rg*^−/−^ mice (lacking mature B, T, and NK cells) are fully susceptible to lethal intranasal HSV-1 or intravaginal HSV-2 infection, respectively (Ashkar and Rosenthal 2003; Zolini et al. 2014). Furthermore, B cell (*Ighm*^−/−^)- , MHC I (*B2m*^−/−^)- , and T cell (*Cd4*^−/−^ and *Cd8*^−/−^)-deficient animals are all more susceptible than WT mice in different models of HSV infection and HSE (Beland et al. 1999; Holterman et al. 1999; Manickan and Rouse 1995; Zolini et al. 2014). As with other viral infections, effector CD4^+^ T cells and cytotoxic CD8^+^ T cells play a major role in the elimination of replicating virus. A loss-of-function mutation in CD45 (*Ptprc*^*L*3*X*^), identified in a chemical mutagenesis screen for HSV-1 susceptibility in mice, results in a lack of T and NK cells that drives susceptibility to HSE (Caignard et al. 2013). Here, CD8^+^ T cells, supported by IFNγ-producing CD4^+^ T effector cells, are required for effective viral clearance in the CNS. In response to infection, CD4^+^ T cell-specific *Stat3*^−/−^ mice have increased HSV-1-specific CD8^+^ T cells that express lower levels of KLRG-1 and IFNγ, suggesting that antiviral CD8^+^ T cell function relies in part on their cooperation with STAT3-competent CD4^+^ cells (Yu et al. 2013). In addition, CD8^+^ T cell expression of Dok-1 and Dok-2 proteins, that each function in proliferation, activation, and migration of hematopoietic cells, has been shown to support and amplify HSV-1-specific CD8^+^ effector responses; the absence of *Dok1* and *Dok2* does not, however, impact viral titers in the cornea or TG during primary infection (Lahmidi et al. 2017). Other CD8^+^ T cell defects have also been found to increase susceptibility, notably in lymphotoxin-α-deficient (*Lta*^−/−^) mice that, without adequate cytotoxic IFNγ^+^CD8^+^ T cell responses, succumbed to lethal intramuscular HSV-1 infection (Kumaraguru et al. 2001).

### Pro-inflammatory cytokines and immune cell invasion

The contribution of the immune system to HSE resistance is further supported by findings that single cytokine gene-knockout models are highly susceptible to infection. While the elevated and concerted expression of pro-inflammatory cytokines is a hallmark of pathological inflammation, individual cytokines are essential and non-redundant in a protective response. At high doses of corneal HSV-1, IFNγ receptor-null (*Ifngr*^−/−^) mice are more susceptible to HSE than both *Ifng*^−/−^ and WT mice (Cantin et al. 1999). Yet, *Ifng*^−/−^ mice are partially susceptible to HSV-1 and HSV-2 infection (Ashkar and Rosenthal 2003; Mansur et al. 2005), and approximately 75% of mice succumb to lower doses of corneal HSV-1 between days 10 to 16 post-infection, despite controlling virus as well as resistant WT mice by day 8 (Ramakrishna and Cantin 2018). This may suggest that IFNγ plays a role in limiting cell infiltration and inflammation, rather than as a direct antiviral mechanism (Ramakrishna and Cantin 2018). Yet in the context of HSV-1 infection, the proliferative and cytotoxic capacities of NK cells and of effector CD4^+^ and CD8^+^ T cells also rely on IFNγ expression to clear HSV-1-infected cells and reduce viral load in the CNS (Caignard et al. 2013).

Furthermore, susceptible *Ifng*^−/−^ mice express elevated levels of granulocyte colony stimulating factor (G-CSF), promoting the excessive CNS recruitment of degranulating neutrophils (Ramakrishna and Cantin 2018). G-CSF also induces the expression of suppressor of cytokine signaling 2 (SOCS2). Validating this pathway, either depletion of G-CSF in *Ifng*^−/−^ mice (Ramakrishna and Cantin 2018) or ablation of SOCS2 (*Socs2*^−/−^ mice) (da Cunha Sousa et al. 2016), lowered neutrophil infiltration and thus increased HSE resistance. As for the repressive function of IL-10, *Il10*^−/−^ mice are highly susceptible to lethal HSE (Ramakrishna and Cantin 2018). IL-6, which is known to be overexpressed in the CNS at the peak of HSE, also confers protection to HSE in IL-6-competent mice compared to *Il6*^−/−^ mice (LeBlanc et al. 1999).

Tumor necrosis factor (TNFα) is another example of a key pro-inflammatory cytokine that is involved in neuroinflammation, and yet also acts to control infection and prevent encephalitis. *Tnf*^−/−^ mice are susceptible to HSE, where HSV-1 disseminates past the brainstem and cerebellum (Lundberg et al. 2007; Sergerie et al. 2007). *Tnf*^−/−^ mice share a similar susceptibility profile with *Il1b*^−/−^ and *Tnf*^−/−^*Il1b*^−/−^ double-knockout mice, which lack pro-inflammatory IL-1β processed downstream of the inflammasome complex and capable of activating lymphocytes and inducing IFN I and II production (Sergerie et al. 2007). IL-1-family cytokine IL-36β (*Il36b* gene) is also involved in protective immunity to lethal HSE (Milora et al. 2017). As with circulating immune cells, TNFα is produced by resident microglia in the brain; its cognate receptors p55 (*Tnfrsf1a*) and p75 (*Tnfrsf1b*), expressed on infiltrating and resident immune cells, play a more complex role in HSV immunity. One study found that a 3200 plaque-forming units (PFU) HSV-1 corneal infection of *Tnfrsf1a*^−/−^ mice resulted in self-resolving viral replication in the brainstem and TG. WT and *Tnfrsf1a*^−/−^*Tnfrsf1b*^−/−^ double-knockout mice were also resistant to HSE (Lundberg et al. 2007). In another study, *Tnfrsf1a*^−/−^ mice were only slightly more sensitive at a higher dose (10^5^ PFU) of corneal HSV-1 than WT mice (Mohankrishnan et al. 2017). Together, these studies suggest that a protective response to HSE depends on TNFα and not its receptors, or otherwise partly relies on p55-mediated inflammation and cytokine production in certain models of acute disease. A final cytokine that contributes to resistance is inducible nitric oxide synthase (iNOS; *Nos2* gene), produced chiefly by myeloid cells. iNOS-deficient mice exhibit poor virus control following footpad HSV-1 infection (MacLean et al. 1998), and 100% mortality to intranasal infection and an overall increase in pro-inflammatory factors TNFα, CCL2, Rantes and CXCL10 in the TG characteristic of HSE (Zolini et al. 2014).

While protective cellular responses depend on the above cytokines, their elevated expression can compromise the integrity of the BBB and allow cells to infiltrate and further damage the infected CNS. In mouse models of neurotropic infection, the infiltration of immune cells is enabled by TNFα and IL-6, and various matrix metalloproteases are known to reduce the expression of endothelial and tight junction proteins at the barrier (Ashley et al. 2017; Li et al. 2015; Rochfort et al. 2016). CNS lymphatics can also facilitate infiltration (Louveau et al. 2015). Following HSV infection, the further development of blood and lymph vessels has been studied in the context of the cornea. TNFα accelerates HSV-1-driven lymphangiogenesis in the corneal epithelium, as does IL-6 in the absence of TNFα (Bryant-Hudson et al. 2014). Here, viral *ICP4* transcripts act as enhancers of vascular endothelial growth factor A (VEGF-A) expression to increases vascularization and HSV-1-specific antiviral CD8^+^ T cells in the cornea and TG (Gurung et al. 2017). The role of lymphangiogenesis is less clear in the brain of HSE-susceptible mice. However, both vascular permeability and lymphoid or myeloid cell CNS infiltration is reduced in platelet activating factor receptor-deficient (*Ptafr*^−/−^) mice, resulting in delayed mortality upon intracranial HSV-1 (Vilela et al. 2016).

### CNS-infiltrating immune cells and microglial activation

In the infected CNS, once thought to be off-limits to the immune system, the activation of resident cells and the infiltration of immune cells can either restrict viral replication to limit inflammation, or contribute directly to lethal encephalitis. Trafficking lymphocytes can be detected as early as day 5 post-HSV-1 infection in the TG, where TLR2- and TLR9-dependent pathways drive the production of granzyme B and perforin by cytotoxic NK and CD8^+^ T cells, of iNOS by macrophages, and of IL-1β by conventional DCs (Lucinda et al. 2017). The infected TG are also noted for their high expression of CCL2 in susceptible *Tlr2*^−/−^*Tlr9*^−/−^ mice, which leads to further recruitment of monocytes and T cells to the CNS. In addition, infiltrating neutrophils and F4/80^+^ macrophages are known to contribute directly to HSE pathology in susceptible 129 background mice (Lundberg et al. 2008).

CXC-motif chemokines secreted by resident glial cells, blood–brain endothelial cells, or hematopoietic cells help to recruit inflammatory cells that express cognate chemokine receptors. CXCL10 expression is sharply upregulated upon infection and, like fellow chemokine CXCL9, binds receptor CXCR3 expressed on monocytes, NK cells, and T cells to promote their homing to the site of infection. Thus, *Cxcl10*^−/−^ mice are more sensitive to lethal HSE than WT mice, and fail to control viral replication in the brain stem due to low recruitment of NK and CD8^+^ T cells (Wuest and Carr 2008). CXCL10 is also implicated the protective response to HSV-1-related pathology in the cornea, and in the absence of CXCL10, CXCL9 may play a compensatory role to limit herpes keratitis (Tajfirouz et al. 2017). Moreover, bone marrow chimeric mouse models have been very useful to limit monogenic defects in chemokine signaling to either the hematopoietic or tissue-resident cell compartments. The HSE-protective effect of CXCL10 is dependent on bone-marrow-derived cells that home to the CNS, and not on radio-resistant microglia or stromal cells of the CNS (Wuest et al. 2011). For example, it has been proposed that CXCR3^+^ NK cells, for which CXCL10 is thought to be the principle attractant, may be important effectors of the antiviral response in *Cxcl10*-competent mice (Wuest and Carr 2008).

Although CXCL10 has a non-redundant protective role in the CNS, mice that lack its receptor CXCR3 (*Cxcr3*^−/−^) are resistant to HSV-1 infection, and slightly more resistant than WT C57BL/6 mice (Wickham et al. 2005; Zimmermann et al. 2017), suggesting that some CXCR3^+^ immune cells may be the cause of lethal inflammation. Consistent with their survival, *Cxcr3*^−/−^ mice maintain control of viral replication in the brain ependyma and hippocampus by relying on intact IFNβ signaling (Kroll et al. 2014; Zimmermann et al. 2017). However, low cytokine (TNFα, IFNγ) and chemokine (CXCL9, CXCL10, CCL2) expression in resistant animals limits leukocyte infiltration (Zimmermann et al. 2017). CXCR3-deficiency also completely excludes activated CD8^+^ T cells from the CNS, cells that otherwise upregulate CXCR3 in WT hosts. Thus, the infiltration of CXCR3^+^ monocytes and T cells from the periphery drives HSE pathology in mice (Menasria et al. 2015; Zimmermann et al. 2017).

Of note, other models of defective chemokine receptor signaling show altered recruitment of immune cells to the CNS without necessarily affecting survival or outcome. For example, *Ccr5*^−/−^ and WT mice are both completely susceptible to intracranial HSV-1, yet knockouts display increased neutrophil homing to the CNS (Vilela et al. 2013). Alternatively, *Ccr5*^−/−^ mice feature higher numbers of T cells in the TG, but are only slightly more resistant to corneal HSV-1 infection compared to WT (Carr et al. 2006). CCR5 receptor is expressed on most immune cells, neurons, and glial cells, and engages many chemokines including CCL3 (MIP-1α), CCL4 (MIP-1β), and CCL5 (RANTES); the expressions of these and other chemokines are all increased in *Ccr5*^−/−^ mice in both infection models, hinting at an intricate compensatory mechanism. Thus, while the role of individual chemokine signaling pathways in protective or detrimental host responses is complex and not fully understood, the excessive infiltration of cytotoxic and inflammatory cells to the CNS is most often auxiliary to HSE pathogenesis.

Finally, CNS-resident microglia can play an important role in the development of HSE, especially in the context of innate antiviral immunity. Abundant macrophage-like glial cells, microglia are activated in the HSV-1-infected brain and can produce a number of chemokines and cytokines including IFN I that can help control viral replication, or in the long-term promote HSE pathology (Conrady et al. 2013; Marques et al. 2008; Wuest and Carr 2008). Expression of CX3CR1 on microglia, which recognizes inhibitory CX3CL1 produced by healthy CNS cells, helps maintain a protective, anti-inflammatory environment in the brain. Therefore, *Cx3cr1*-deficiency in the radio-resistant compartment of chimeric mice is sufficient to render them susceptible to lethal intranasal HSV-1 infection (Menasria et al. 2017). Here, *Cx3cr1*^−/−^ microglia fail to contain HSV-1 spread in the CNS, which recruits elevated numbers of neutrophils and inflammatory monocytes to the site of infection. Furthermore, the ablation of CD118 (IFNAR1) in radio-resistant microglia has been shown to drive HSV-1 viral load in the CNS, implicating TRIF-dependent production of IFN I by microglia in the protective response to HSE (Conrady et al. 2013). Ultimately, microglia are required for proper IFN I-mediated control of HSV-1 in infected neurons and glial cells of the CNS, and to avoid a general shift towards lytic cell death and neuroinflammation that underlie HSE.

## Cell death and autophagy in mouse HSE pathology

Recent studies have also relied on mouse models and primary cells to investigate cell death pathways and their role in the pathology of the infected CNS. In the brain, homeostasis is maintained by patrolling microglia that detect pathogens and damage signals, while the more numerous astrocytes promote neuronal survival. In primary murine cultures, HSV-1 infects both these glial cell types, and both secrete pro-inflammatory factors and IFN I (Aravalli et al. 2005). Virus replication is controlled and self-limited in microglia that preferentially undergo early caspase 3- and TNF-dependent apoptosis; infected astrocytes yield higher viral titers and die at later time-points from Fas-dependent apoptosis (Aravalli et al. 2006).

In the context of the CNS, apoptotic cell death can function as a cell-intrinsic antiviral mechanism by depriving the virus of host cells within which to replicate, but can also be a consequence of infection that drives pathology and inflammation. For example, cells lacking X-box binding protein 1 (*Xpb1*^−/−^), a key player in the unfolded protein response that relieves stress at the endoplasmic reticulum, are unable to initiate apoptosis upon HSV-1 infection, resulting in more elevated viral titers (Fink et al. 2017). More generally, various cellular stress responses, including infection, can signal through p53 to promote cell death and apoptosis. Following *in vitro* HSV-1 infection, p53 can enhance or depress the expression of viral proteins ICP22 and ICP0, respectively, to modulate viral replication in a human epithelial cell line (Maruzuru et al. 2013). In vivo, p53-null (*Trp53*^−/−^) mice are significantly more resistant to intracranial HSV-1 infection than susceptible WT mice (Maruzuru et al. 2016). Further, the p53-deficient CNS exhibits no important cell death or HSE pathology, and reduced infiltration of immune cells, suggesting that virus is controlled independently of cell death; p53-dependent gene signatures are associated with HSE in susceptible *Trp53*^*+/+*^ control mice. Thus, apoptosis of infected glial cells, and likely including infected neurons, appears to be detrimental and central to HSE pathogenesis in mice.

Neurons are especially vulnerable to acute neurotropic viral infections. Damage caused by lytic neuronal cell death can have severe impact on the host. Consequently, neurons respond very differently from glial cells to IFN I signals, and aim to avoid IFN I-induced apoptosis. Upon infection in vivo, neuron-specific STAT1-deficiency (*Stat1*^*N*−/−^) leads to high viral titers in the TG and brain stem and the development of HSE (Rosato et al. 2016). Murine neurons also do not upregulate apoptosis in response to IFN unlike fibroblasts (Rosato et al. 2016; Yordy et al. 2012). Rather, STAT1-dependent IFNβ signaling only managed to restrict the replication of a mutant Δγ34.5 HSV-1 virus lacking viral protein ICP34.5 (Rosato and Leib 2014). While ICP34.5 expression is known to reduce the activity of host IFN I (Manivanh et al. 2017), these data also support a role for IFN I signaling in initiating antiviral mechanisms alternate to apoptosis, namely, IFN I-induced autophagy.

Viral ICP34.5 has been extensively studied in the context of its Beclin-binding domain, which can interact with Beclin 1 to prevent autophagy in infected neurons (Orvedahl et al. 2007). Thus, neurotropic HSV viruses are equipped to counter autophagy, a well-established recycling mechanism that is preferentially induced in neurons to reduce viral replication while avoiding pathological cell death and ensuring survival (Yakoub and Shukla 2015). Neurons lacking key autophagy factor ATG5 (*Atg5*^−/−^) yield higher viral titers in culture, following infection with both a Beclin-binding domain-deficient and functional HSV-1 virus, suggesting that while ICP34.5 certainly reduces autophagy, autophagy can also be initiated at early time-points before ICP34.5 is expressed to help reduce viral load (Yordy et al. 2012). Alternatively, *Sting*^−/−^ mice are also susceptible to intracranial and corneal infection with Beclin-binding domain-deficient HSV-1 compared to resistant control mice, highlighting the role of STING-dependent autophagy in in vivo antiviral control (Parker et al. 2015). Finally, if not to induce apoptotic cell death or traditional antiviral responses, IFN I does induce the formation of unique autophagosome clusters in treated neurons that are absent in IFN signaling-deficient cells (*Irf3*^−/−^, *Ifnar*^−/−^*Ifngr*^−/−^, *Stat1*^−/−^) (Katzenell and Leib 2016). As autophagy is less impeded in infections with Beclin-binding domain-deficient HSV-1, the mutant virus also spreads differently in the BALB/c brain, where increased autophagy and NLRP3-dependent inflammasome activation correlated with a reduction in viral burden in the brain and lower HSE incidence (Zhang et al. 2013a). Thus, these data suggest that neurons can rely on the IFN I/autophagy axis to control viral replication without resorting to apoptotic cells death, but the efficient blocking of these pathways by viral proteins further complicates our understanding of HSE development in vivo.

## Perspectives

Altogether, mouse models have refined our understanding of HSV infection and invasion of the CNS. Reverse genetic approaches have not only confirmed several childhood HSE-protective genes (*Tlr3, Trif, Stat1*), or revealed others belonging to overarching TLR pathways (*Myd88, Irf3, Irf7, Tlr9*), but have also implicated many innate and adaptive immunity genes that better define mechanisms underlying HSE. Further, genetic mouse models that are differentially susceptible, based on the route of viral entry to the CNS, highlight the importance of early antiviral control by different viral pattern recognition mechanisms at mucosal surfaces, in the TG or in the CNS proper. Accordingly, antiviral treatment with acyclovir is the first-line treatment for HSE in humans, and works in mice to improve survival (Long et al. 2011; Quenelle et al. 2018). IFNα is also capable of limiting HSV infection in human neuronal culture (Pourchet et al. 2017), and is sometimes used in combination therapy to treat other viral infection (Sagnelli et al. 2017). Treatment of mice with cyclic dinucleotide agonists of the cGAS/STING pathway protected animals from HSV-2 replication, while TLR agonist treatment in mice also limited HSE development (Boivin et al. 2008; Skouboe et al. 2018); topical TLR7 agonist imiquimod has been shown to be ineffective in human HSV-2-infected patients (Schacker et al. 2002). In mice under the *Tlr3*-independent IFN I pathway, drug-targetable genes might also include deubiquitinases (TRIM14, USP13, USP21, iRhom2) that modify factors in the cGAS/STING cascade to effect IFN I production and HSE outcome.

These mouse studies have also highlighted an important contribution of trafficking and infiltrating immune cells to HSE pathogenesis. For example, peripheral and CNS-invading NK cells are involved in several gene-knockout models (*Irf9, Ifng, Ifngr, Il15, Cxcl10*); the protective antiviral function of NK cells in murine HSE may warrant further study into using NK cells as a targeted antiviral therapy. In general, cytokines appear to be protective in mouse HSE, despite being involved in late-stage neuroinflammation. Chemokine dynamics play complex roles in limiting or promoting CNS invasion, or even activating resident glial cells. Understanding the balance between inflammation and cell death in the CNS might help to reduce the risk of long-term sequelae that may develop in many cases of acyclovir-treated encephalitis.

Furthermore, these aspects of mouse HSE might help inform mechanisms that underlie different types of viral encephalitis in humans, which are challenging to model appropriately in mice. While neonate and adult mice react differently to CNS infection, current mouse models do not capture the individual variation in human HSE pathologies, which may depend on the neurovirulence of the viral strain, on the primary site of infection, or on the reactivation of HSV from latency in sensory neurons. Other types of encephalitis, including autoimmune anti-NMDAR (N-methyl-D-aspartate receptor) encephalitis, have been reported in older adults that have recovered from HSE earlier in life, but later develop neuroinflammation due to anti-NMDAR antibodies in the cerebrospinal fluid that may stem for exposure to HSV antigens (Armangue et al. 2014; Omae et al. 2018). Viral escape from latency can also be triggered by certain drugs, most strikingly by natalizumab, approved for treatment of relapsing-remitting multiple sclerosis and Crohn’s disease, which has been reported to drive John Cunningham virus (JCV) reactivation from latency in the brain and lead to progressive multifocal leukoencephalopathy in some patients (Bloomgren et al. 2012).

Cases of HSE worldwide are usually reported at 2 to 4 per million individuals per year (Jorgensen et al. 2017). While HSV-1 and HSV-2 prevalence are decreasing in the United States (McQuillan et al. 2018), the ubiquity of these viruses has prompted many studies on the involvement of herpes viruses in complex CNS neurodegenerative or chronic inflammatory diseases. Most notably, HSV-1 infection has been linked to Alzheimer’s disease and to the expression of the apolipoprotein 4 (APOE-ε4) allele isoform (Itzhaki et al. 1997; Steel and Eslick 2015). Besides host genetics, HSV infection is known to increase as people age, and women are twice as likely to be infected with HSV-2; both age and gender are known to affect risk for certain neurodegenerative and chronic inflammatory diseases (McQuillan et al. 2018). Given these important genetic, environmental and pathogen factors, mouse models will continue be useful to dissect the cells and pathways that are implicated in HSV infections of the CNS. As novel techniques are applied to study human HSE in rare patients, including induced pluripotent stem cell differentiation of patient cells to CNS cells (Lafaille et al. 2012; Pourchet et al. 2017; Zhang et al. 2018), we expect that these essential genes identified in animal models and human patients will together help to define mechanisms of pathogenesis and viral control, and better clarify how a widespread and successful viral pathogen only seldom results in lethal encephalitis.
